# The Preliminary Assessment of New Biomaterials Necessitates a Comparison of Direct and Indirect Cytotoxicity Methodological Approaches

**DOI:** 10.3390/polym14214522

**Published:** 2022-10-25

**Authors:** Milena Chraniuk, Mirosława Panasiuk, Lilit Hovhannisyan, Sabina Żołędowska, Dawid Nidzworski, Lidia Ciołek, Anna Woźniak, Zbigniew Jaegermann, Monika Biernat, Beata Gromadzka

**Affiliations:** 1 Department of In Vitro Studies, Institute of Biotechnology and Molecular Medicine, Kampinoska 25, 80-180 Gdańsk, Poland; 2 Biomaterials Research Group, Ceramic and Concrete Division in Warsaw, Łukasiewicz Research Network-Institute of Ceramics and Building Materials, Cementowa 8, 31-983 Kraków, Poland

**Keywords:** biomaterial toxicity testing, chitosan-bioglass biocomposite, direct and indirect toxicity tests, biomaterial evaluation

## Abstract

Background: Cytotoxicity testing is a primary method to establish the safety of biomaterials, e.g., biocomposites. Biomaterials involve a wide range of medical materials, which are usually solid materials and are used in bone regeneration, cardiology, or dermatology. Current advancements in science and technology provide several standard cytotoxicity testing methods that are sufficiently sensitive to detect various levels of cellular toxicity, i.e., from low to high. The aim was to compare the direct and indirect methodology described in the ISO guidelines UNE-EN ISO 10993-5:2009 Part 5. Methods: Cell proliferation was measured using WST-1 assay, and cytotoxicity was measured using LDH test kit. Results: The results indicate that the molecular surface of biomaterials have impact on the cytotoxicity and proliferation profile. Based on these results, we confirm that the indirect method does not provide a clear picture of the cell condition after the exposure to the surface, and moreover, cannot provide complete results about the effects of the material. Conclusions: Comparison of both methods shows that it is pivotal to investigate biomaterials at the very early stages using both indirect and direct methods to access the influence of the released toxins and surface of the material on the cell condition.

## 1. Introduction

Bone regeneration is a complex physiological process in which sequential cellular and molecular events take place to generate a new bone [1,2], rather than a fibrous scar-like tissue. Bone fractures are common injuries that occur continuously throughout adult life that are healed during normal bone remodeling processes [3,4]. Severe bone fractures, bone defects due to trauma and to other complex clinical conditions in which bone regeneration is required in large quantity, cannot fully rely on natural bone healing and require additional support [5,6]. Large bone defects due to skeletal reconstruction, infection, tumour resection, or skeletal abnormalities especially in cranial, oral, and maxillo-facial and orthopaedic surgery are one of the central clinical issues in regenerative medicine [7,8]. There is a high demand to develop new, improved strategies to support the impaired or ‘’insufficient’’ bone-regeneration process [9,10]. One of the most promising methods to improve bone healing is implantation using regenerative biomaterials [11,12].

Many different types of biomaterials are considered for tissue engineering and bone healing, e.g., polymers (polylactide, polycaprolactones, chitosan), hydroxyapatite-based ceramics, bioglasses, etc. [13,14]. Biocompatibility is one of the main prerequisites for the clinical use of biomaterials. Biocompatible materials that support bone regeneration should not only support faster reconstruction of bone tissue [15], but also show no cytotoxicity [16], which can be assessed in vitro using a variety of different cellular models. Usually, assessing the cytotoxic or pro-regenerative effect of individual substances do not cause major difficulties, as most of them are water-soluble. But in case of multi-component solid composites, such as chitosan-bioglass composites presented in this study, a different experimental approach is necessary [17,18,19]. To fully characterize and assess the biocompatibility of such composites it is crucial to consider each constituent substance, as well as the potential mechanical damage to the cells caused by a contact with the whole material. 

Guidelines for testing solid products for future medical use are described in the ISO 10993-5:2009 Part 5 standard [20], which presents two types of material testing: the indirect method and the direct method. The indirect method involves preparation of extracts from material samples which are then incubated with the cells [21]. Therefore, using this method only the effect of substances (including potential toxins) released into the extractant is assessed. In the indirect method it is not important how the surface of the tested material (e.g., roughness, porosity, viscosity) can impact on cell condition and survival [22]. One of the basic assumptions of the direct method is the contact/interaction of cells with a material with at least one flat surface. In this method the condition of the cell depends on both surface structure and substances released into the environment in which cells are grown [23]. When choosing the direct method for biomaterial research it is necessary to take into account the possibility that obtained results are the sum of the previously described phenomena and may differ from results obtained in the indirect method [24]. Moreover, in vitro studies are based on monolayer assay which can also have an impact in case of direct method.

To properly assess the biological properties of the final material it is necessary to select an appropriate cell model and cytotoxicity testing method. For chitosan-bioglass composites used for bone regeneration no broader work has been carried out with the use of healthy osteoblasts, including the human foetal osteoblasts hFOB 1.19 line (Table 1). 

In this original paper we present a comprehensive experimental approach to test the cytotoxicity of solid biomaterials using chitosan-bioglass solid biocomposites as an exemplary research material. The toxicity of chitosan-bioglass composites was examined using hFOB 1.19 cell line by both direct and indirect methods described in the ISO 10993-5:2009 standard.

## 2. Materials and Methods

### 2.1. Chitosan-Bioglass Composites as an Exemplary Research Material

Porous composites developed as materials for bone tissue regeneration were used in this research. Composites were made by the thermal phase separation method based on natural polysaccharide-chitosan. The commercial product Chitoceuticals from Heppe Medical Chitosan (Halle, Germany) was used.

Three bioglasses (P5, P5Sr2, P5Zn2) produced by the sol-gel method were used as a filler for the developed composites. Bioglasses were made using the SiO_2_-P_2_O_5_-CaO system. The chemical composition of the P5 bioglass included: 70% wt. SiO_2_, 5% wt. P_2_O_5_, and 25% wt. CaO. Bioglasses P5Sr2 and P5Zn2 were enriched with a share of 2% wt. SrO or 2% wt. ZnO instead of 2% CaO, respectively.

Seven composites differing in the type of bioglass and their content were used for the tests: CHBG1, CHBG2a, CHBG2b, CHBG2c, CHBG3a, CHBG3b, CHBG3c. Composites marked with a, b, and c had a polymer to bioglass ratio of 1:0.5; 1:1; 1:2, respectively (Table 2).

### 2.2. Characteristics of Composites Microstructure by Field Emission Scanning Electron Microscopy (FE-SEM)

The microstructure of the obtained porous composites was tested by scanning electron microscopy with field emission (Nova NanoSEM 200, FEI, Amsterdam, Holand). Briefly, the samples were covered with a conductive material (25 nm gold film) using a sputter coater (EM SCD500, Leica, Wien, Austria). Imaging of composites was visualized in high vacuum conditions using an ETD detector (Everhart–Thornley detector combined with Nova NanoSEM 200) at 10 kV accelerating voltage and at magnifications of 500×. On the basis of composites, pictures obtained by microscopy pore sizes was measured. At least 16 measurements of each composite pores were collected. Obtained data were used in statistical analysis and hierarchical clustering.

### 2.3. Cell Culture

Immortalized human foetal osteoblastic cell line (hFOB 1.19 cells; ATCC nr CRL-11372, Rockville, MD, USA) was cultured in 1:1 mixture of Ham’s F12 Medium and Dulbecco’s Modified Eagle’s Medium with 2.5 mM L-Glutamine (without phenol red; Thermo Fisher, Waltham, MA, USA). The medium was supplemented with 10 µg/mL of gentamicin and 0.25 µg/mL amphotericin B (Thermo Fisher, USA) and Foetal Bovine Serum (FBS; Thermo Fisher, USA) at the final concentration of 10%. Cells were cultured at 34 °C and 5% CO_2_ (according to ATCC culturing protocol for hFOB 1.19) in CB240 incubator (Binder, Tuttlingen, Germany). The above-mentioned cell culture medium was used in the experiments mentioned. hFOB 1.19 cell line was obtained from LGC Standards (Kiełpin, Poland).

### 2.4. Direct Contact Method

Prior to the experiment the composites were inserted into 24-well plates (Sarstedt, Nimbrecht, Germany) and immersed in 1 mL of culture medium for 1 h. Sterilized Raschig rings made of borosilicate glass 3.3 (Simax, Praha, Czech Republic) were placed on the top of the composites, followed by hFOB 1.19 cell seeding. In each well 5 × 10^4^/cm^2^ hFOB 1.19 cells were seeded inside the Raschig ring.

Plates were incubated for 1 h at 34 °C and 5% CO_2_ in CB240 incubator (Binder, Germany) allowing the cells to attach to the composites. After 1 h, rings were removed, and the cells were incubated for 48 h. After that time the cell viability was estimated by measuring the mitochondrial activity (WST-1 test) and the cell cytotoxicity was measured by the leakage of lactate dehydrogenase (LDH cytotoxicity assay). All types of the composites were tested in at least 4 replicates.

### 2.5. Indirect Contact Method

Different composites were inserted into 24-well plates (Sarstedt, Germany) and immersed in 1.1 mL culture medium for 24 h at 34 °C and 5% CO_2_ in CB240 incubator (Binder, Tuttlingen, Germany). After the incubation, the extract was collected. The day prior to the experiment, the hFOB 1.19 cells were seeded at the density of 5 × 10^4^/cm^2^ on 24-well plates. On the next day, the conditioned media was replaced with the extracts, and the cells were incubated for 48 h at 34 °C and 5% CO_2_. After the incubation, cell viability was estimated by measuring the mitochondrial activity (WST-1 test) and cell cytotoxicity was measured by the leakage of lactate dehydrogenase (LDH cytotoxicity assay). All types of the composites were tested in a least 4 replicates. To obtain the extract, each tested sample was prepared from one disc.

### 2.6. Water Soluble Tetrazolium Salt-1 (WST-1) Cell Proliferation and Mitochondrial Activity Assay

The proliferation of hFOB 1.19 cells was determined by using WST-1 assay kit (Abcam, Cambridge, UK) according to the supplier’s protocol. Briefly, the cells were incubated with the extract or composites then treated with 40 µL of WST-1 reagent followed by 2 h incubation at 34 °C and 5% CO_2_.

Conditioned media was collected from each well and transferred to a 96-well flat bottom plate (Sarstedt, Germany). The optical density at 450 nm and 620 nm was measured using a plate reader Epoch (BioTek Instruments, Winooski, VT, USA). Untreated cells, blank medium, and the control of composite sterility were included into each assay. Percentage of proliferation was calculated as follows:

Proliferation [%] = (Sample absorbance/Control absorbance) × 100%(1)

All controls in calculations were means of tetraplicates.

### 2.7. Lactate Dehydrogenase (LDH) Cytotoxicity Assay 

Membrane integrity and viability of hFOB 1.19 cells was measured by estimating the leakage of lactate dehydrogenase (LDH) from the cells incubated either with composites directly or with the extract. The test was performed using the Cytotoxicity Detection Kit^PLUS^ (Roche Applied Science, Mannheim, Germany) according to the supplier’s protocol. Briefly, the dye solution was mixed with the catalyst solution and added to the samples. After incubation in the dark, the optical density at 490 nm and 690 nm was measured using a plate reader Epoch (BioTek Instruments, Winooski, VT, USA).

Positive control cells treated with Triton-X100 solution (included in the kit) were used. Untreated cells, blank medium, and the control of composite sterility were included into each assay. Percentage of the cytotoxicity was calculated as follows:

Cytotoxicity [%] = (Sample absorbance − Control absorbance)/(Positive control absorbance − Control absorbance) × 100%

All controls in calculations were means of tetraplicates.

### 2.8. Statistical Analysis

Collected data were analysed and visualised using GraphPad Prism (GraphPad Software, San Diego, CA, USA). Due to the limited number of experimental samples, the normality of results’ distribution could not be confirmed. Thus, statistical calculations for different amounts of data obtained in the experiments were performed using the Mixed-effects Model, which is based on Restricted Maximum Likelihood (REML) calculations (*p* = 0.05). In the next step, to control the false discovery rate, the Benjamini, Krieger, and Yekutieli multiple comparison test (*p* = 0.05) was carried out. Results accumulated from the experiments using direct and indirect methods were compared for all composites in two groups—cytotoxicity and proliferation. Cytotoxicity and proliferation data are presented as means with standard deviation. 

Before the main statistical analysis of pore sizes, outlier values within collected data were identified based on the False Discovery Rate (FDR) with Q = 1%. One outlier value was found in the CHBG3c dataset and was excluded from further analyses. Due to the sufficient number of pore size measurements, a panel of normality and lognormality tests was carried out to confirm the type of data distribution. Both types of data distribution were detected, therefore statistical comparison of the pore sizes was conducted using the Kruskal–Wallis test (*p* = 0.05) and Dunn’s multiple comparisons test (*p* = 0.05). Pore size distribution data are presented as a box and whiskers graph with the median, 25th and 75th percentile as boxes, and the minimum and maximum values of pores sizes as whiskers.

### 2.9. Hierarchical Clustering

Collected data were analysed and visualised using NCSS Software (NCSS LLC, Kaysville, UT, USA). Hierarchical clustering was conducted using the full dataset obtained in this research which includes: median of pores sizes, averages of proliferation, and cytotoxicity in the direct and indirect methods, chitosan-bioglass ratio in composites, and amounts of oxides in bioglass. Group Average (Unweighted Pair-Group) was chosen as clustering method, and the Euclidean method was used in distance calculations with a standard deviation scaling method. Results of hierarchical clustering are presented as a dendrogram.

## 3. Results

### 3.1. Characteristics of Composites

**Figure 1:**
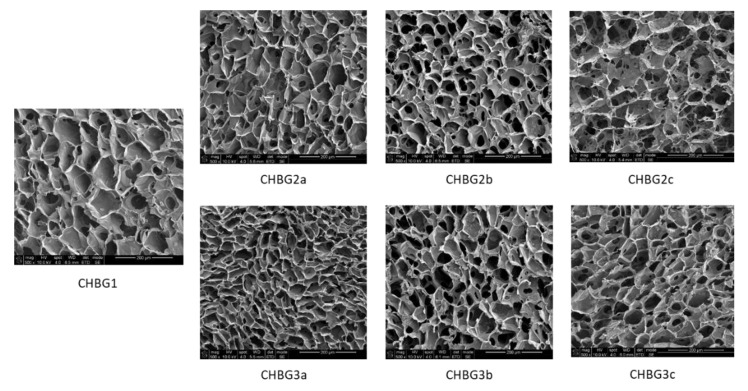
Microstructure of porous composites used in direct and indirect toxicity tests. Porosity was observed by electron microscopy with field emission (FE-SEM).

**Figure 2:**
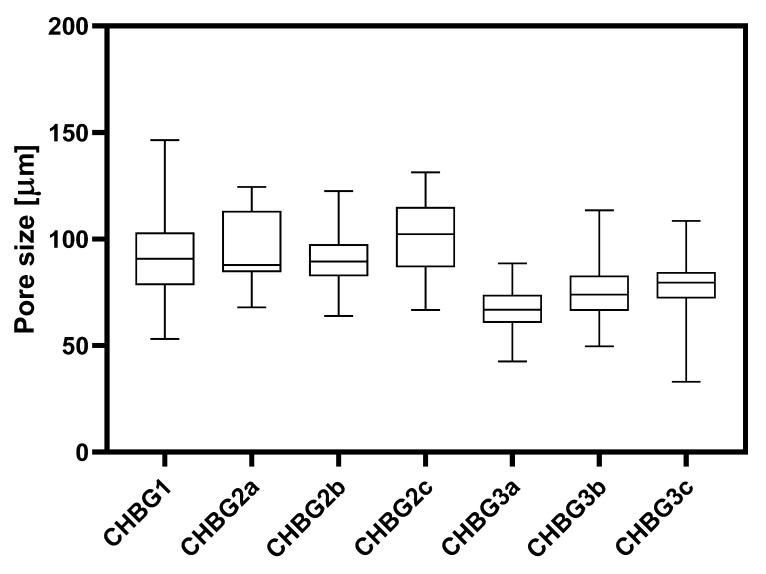
The distribution of pore sizes of the composites used. Data are presented as box and whiskers, with boxes representing the median and 25th to 75th percentiles, and whiskers as maximum and minimum values of pore sizes. Statistically significant differences (*p* < 0.05) between pore sizes are presented in the Supplementary Table S1.

Chitosan-bioglass composites are porous chitosan scaffolds produced by the freeze-drying method with different contents of two types of bioglass embedded in the polymer matrix in the pore walls. All composites were characterized by open porosity which allowed for the free flow of nutrients and the pore size in the range of 33–146 µm. Due to the fact that during the freeze-drying process solid particles may constitute points of nucleation, their different content in the composition results in a different porosity [32]. As a rule, the more bioglass grains, the smaller the pores are formed. As the polymer content increases in relation to the amount of bioglass, pores are formed less but they are larger, which is confirmed by our observations (Figure 1 and Figure 2).

Statistically significant differences between pore sizes were detected for the base composite CHBG1 with CHBG3a (*p* < 0.0001) and CHBG3b (*p* < 0.0001). The pore sizes were not statistically different for all CHBG2 composites. There were also no statistically significant differences between the pore sizes of CHBG3 composites and between CHBG3c and CHBG1. Comparisons of pore sizes between analogous composites with ZnO or SrO showed the presence of statistically significant differences between CHBG2a vs. CHBG3a (*p* < 0.0001) and CHBG3b vs. CHBG2b (*p* < 0.0001). The full data obtained during the statistical analysis is presented in the Supplementary Table S1.

### 3.2. Cell Proliferation

Proliferation of cells seeded directly on bioglass-chitosan composites (direct method) or cells incubated with an extract (indirect method) was measured using the WST-1 assay, which is based on the mitochondrial activity. All results were calculated as a % of control cells (arbitrary set as 100%). Both methods were performed according to the ISO 10993-5:2009 guideline. 

**Figure 3:**
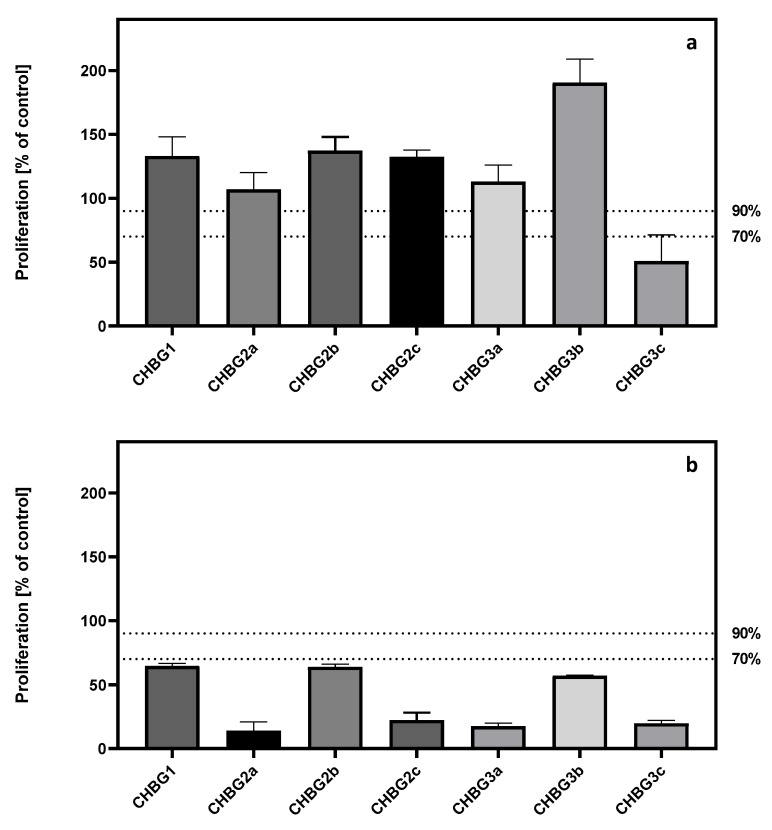
Proliferation of hFOB 1.19 cells after 48 h of incubation with (**a**) chitosan-bioglass extracts in the indirect method, (**b**) composite in the direct method. Cell proliferation was measured using WST-1 assay. All results were calculated as a % of control cells (arbitrary set as a 100%). CHBG1, CHBG2a, CHBG2b, CHBG2c, CHBG3a, CHBG3b, and CHBG3c porous composites developed for bone tissue regeneration were used as a model material in this research. Statistically significant differences (*p* < 0.05) between pore sizes are presented in the Supplementary Tables S2 and S3.

Incubation of hFOB 1.19 cells with extracts of composites incubated with cell medium for 24 h revealed that 6 out of 7 extract types increased the mean mitochondrial activity (cell proliferation). The mean values of proliferation exceeded the threshold of 70%, which according to ISO 10993-5:2009 is the limit value below which the extract or material is considered toxic. Additionally, a threshold of 90% was introduced, which is an indicator of the first toxic effect. In the indirect method, only the extract of CHBG3c material did not induce proliferation higher than 90% (Figure 3a).

For measurements performed on cells seeded directly on the composites, we observed that none of the composites allowed cells to grow more than 90% compared to the control cells that didn’t have a contact with the composite. Moreover, the proliferation of cells growing on the composites did not exceed 70% of the control. The highest values of proliferation (>50%) were detected in the cells that had a contact with CHBG1, CHBG2b, or CHBG3b (Figure 3b).

The comparison of proliferation results observed after incubation with the extracts (indirect method) and composites (direct method) indicated the presence of statistically significant differences between the test methods for each composite (for each comparison *p* < 0.0001).

Particularly, noteworthy is the lack of statistically significant differences in the indirect method between the effects of extracts from composites CHBG2c and CHBG1, CHBG2c and CHBG2b, CHBG2a and CHBG3a, and CHBG1 and CHBG2b (Supplementary Table S2). In the direct method, no significant differences are detected for 9 out of 21 comparisons between composites (Supplementary Table S3).

### 3.3. Cell Cytotoxicity

The cytotoxic effect of extracts and composites on hFOB 1.19 cells was tested using the LDH test, which is based on the lactate dehydrogenase activity released from the cytoplasm into the environment through damaged cell membrane. 

**Figure 4:**
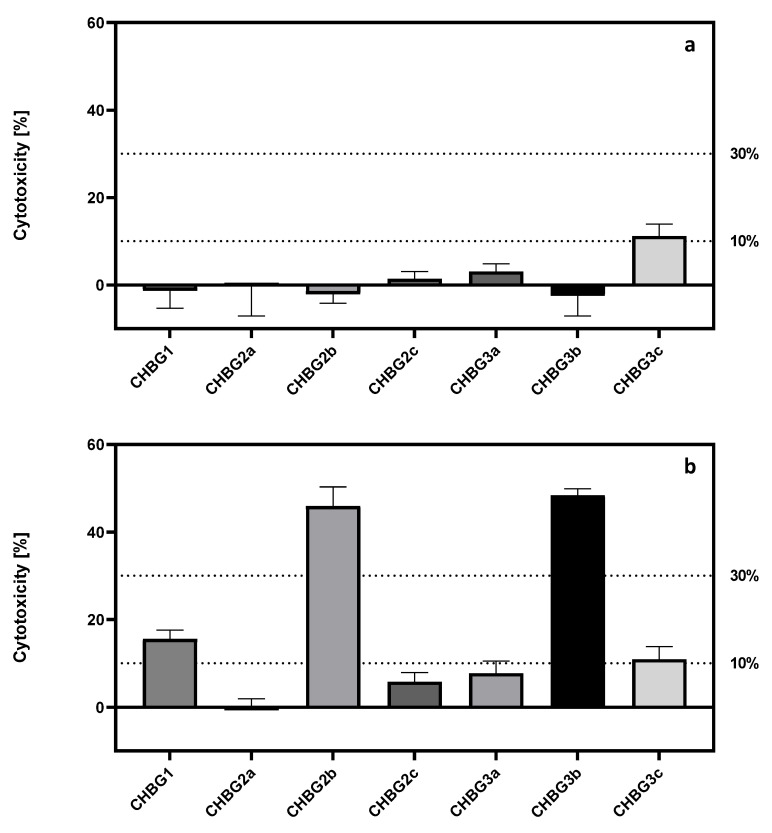
Cytotoxicity of chitosan-bioglass (**a**) extracts in the indirect method, (**b**) composite in the direct method tested after 48 h of incubation with hFOB 1.19 cells. Cytotoxicity was measured using LDH assay. Results were calculated as a % of positive control cells (cells treated with 1% Triton-X100). CHBG1, CHBG2a, CHBG2b, CHBG2c, CHBG3a, CHBG3b, and CHBG3c porous composites developed for bone tissue regeneration were used as a model material in this research. Statistically significant differences (*p* < 0.05) between pore sizes are presented in the Supplementary Tables S5 and S6.

Our results show that the cytotoxicity measured by indirect method did not exceed 30% (Figure 4a). According to ISO 10993-5, this value is considered to be the threshold value above which the material is considered toxic. Among the extracts, there was only one average cytotoxicity value exceeding 10%, which was taken as the indicator of the first toxic effect. In the direct method only 3 composites did not cause a cytotoxic effect exceeding 10% (CHBG2a, CHBG3a, and CHBG2c) (Figure 4a).

Moreover, in the direct method, incubation of cells on CHBG2b and CHBG3b composites resulted in mean cytotoxicity values exceeding 40% (Figure 4b).

Similar to the previous statistical analysis for the data obtained in proliferation tests, we observed a statistically significant (*p* < 0.0001) difference between the direct and indirect methods of testing composites. Further comparisons of the cytotoxicity results observed after incubation with the extracts and directly with the composites indicated the presence of statistically significant differences for 5 out of 7 composites (*p* < 0.0001 for each comparison) (Supplementary Table S4).

For CHBG3c and CHBG2a, no statistically significant differences were found between the direct and indirect tests.

At a further stage of the statistical analysis, the cytotoxicity comparison was made between individual composites in the direct method and between the effect of extracts on damage to the cell membrane.

There were no statistically significant differences among the data collected in the indirect method for 8 out of 21 comparisons of the effect of extracts (Supplementary Table S5). Comparisons of the cytotoxicity values caused by the contact of cells with the molecular surface of composites in the direct method revealed statistically significant differences for each of the compared effects (Supplementary Table S6).

### 3.4. Hierarchical Clustering

Experimental data obtained via indirect and direct testing methods may differ significantly. Further improvements of materials based on those results should be focused on many details, such as the composition, structure and biological response. Then, many variables are involved in selecting the best parameters the analysis of the results obtained in both testing methods, and statistical data processing is necessary. However, these steps may be insufficient for choosing the best type of material or group with similar features, especially when the composition and research results represent high similarity. Hierarchical clustering based on the analysis of multiple datasets collected can be successfully used to correlate multiple variables to reduce the redundancy of information and highlight similarities between composites. This approach can also highlight previously invisible connections between variables and facilitate the optimisation leading to the best possible combination of features of the material.

**Figure 5:**
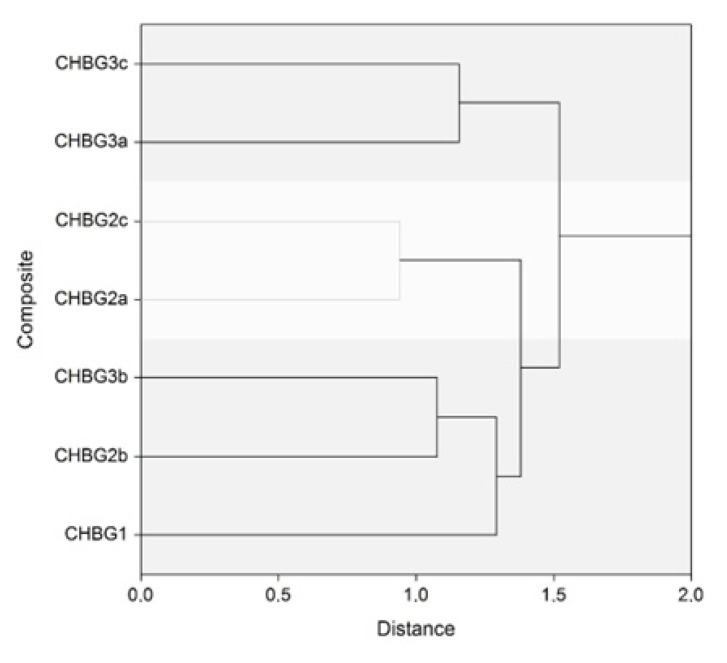
Dendrogram for composites based on data obtained in the research: median of pores sizes, averages of proliferation and cytotoxicity in direct and indirect method, chitosan-bioglass ratio in composites, and amounts of oxides in bioglass.

Considering the physicochemical properties and the biological activity of the composites, the highest similarity was found between the CHBG2b and CHBG2c (Figure 5). Close relation was also found for the group of composites CHBG2b, CHBG3b, and CHBG1, which had the same ratio of chitosan and bioglass. Another group of the composites was detected for CHBG3a and CHBG3c composites. This group formed the most distant cluster in relation to the other composites.

## 4. Discussion

One of the most important features of all biomaterials is their biocompatibility. To fully determine the properties of the new material, it is necessary to conduct not only physicochemical, but also biological tests. To uniformly test the in vitro cytotoxicity of future medical devices, the ISO 10993-5:2009 standard was created. This document states that materials can be tested using direct, indirect, or both methods. 

This paper is a comprehensive study of exemplary research material (chitosan-bioglass composites) supporting bone regeneration, and presents results of the direct and indirect toxicity testing. Results obtained using both methods show statistically significant differences between them and support the hypothesis that the method of testing may affect the results. Our study indicates that the experimental methodology and set up needs to be tailored towards the scientific hypothesis. 

The direct method involves seeding cells directly on the surface of the tested material. It is especially recommended when the intended use of materials and products is associated with their longer contact with tissues. In this method, both the influence of substances secreted by the product and the interaction of cells with the surface of the material are tested. Indirect method of cytotoxicity testing includes the preparation of extracts of the tested materials, and is especially recommended when the product does not have flat surfaces on which cells can be deposited [33,34] or has other features making the direct method not feasible, e.g., a density lower than that of the culture medium. In this method, only the influence of secreted substances is tested on the cell model. 

Assessing the cytotoxic or pro-regenerative effect of multi-component solid biomaterials used for bone regeneration, such as porous chitosan-bioglass composites, may be challenging. Not only the substances released from the material, but also the structure of the material’s surface can have an influence on the condition and survival of cells. Due to those reasons, obtained results may vary between the direct and indirect methods [35,36]. 

The direct method is an attempt to recreate in vivo conditions in which cells are in prolonged contact with the material. However, it is necessary to bear in mind that in vitro tests have their limitations as they are carried out on cell cultures that form a monolayer. In vivo the medical device will interact with multi-layered tissues, and the prolonged, direct contact with the material may affect the response of the body’s immune system [37]. Additionally, in the direct method, the locally increased concentration of the substances released from the material needs to be taken into consideration. The described phenomena may cause higher cytotoxicity and lower cell proliferation. It is very important to remember that the contact of cells with released substances is variable because their concentration changes over time due to diffusion and other processes, such as degradation of the material. For some materials and devices, the effect of substances released during contact with the human body may be much more important than the structure or surface supporting an appropriate environment for cell growth or tissue regeneration [38]. 

The cytotoxic effect of the examined material can be assessed on the basis of results obtained from rapid tests which use different biological activity parameters e.g., mitochondrial activity, ATP synthesis, damage to the cytoplasmic membrane, the ability to uptake or remove dyes from cells. Results obtained from the test measuring survival and proliferation of the cells (such as tests based on mitochondrial activity e.g., WST-1, MTT, XTT, Alamar Blue, and LDH) should be complementary, and ideally, obtained values should add to 100% between both tests. In this study, such situation occurred for the composites CHBG3b and CHBG2b tested by direct method. Death of more than 40% of cells caused by contact with composites and detected in the LDH test coincides with the reduced to approximately 60% proliferation determined in the WST-1 test. However, for the rest of the composites tested by direct method, values obtained in both tests did not add up to 100%, which suggests that additional phenomena, apart from the cell membrane perforation, occurred. For the results obtained by indirect method of testing, high cell proliferation and low cytotoxicity was measured for most materials tested. Differences between results obtained by direct and indirect methods suggest the need for further tests to assess the physiological state of the model as well as the determination of the likely impact of the material structure on cells.

Cluster analysis revealed similarity between the CHBG1, CHBG2b, and CHBG3b composites. Despite detecting statistically significant differences between the pore size of the base composite and the ZnO-enriched composite, as well as the pore size of CHBG2b and CHBG3b, the response of the cell model to tested materials or their extracts was comparable in both indirect and direct methods. Based on this information, we conclude that the size of the pores and/or the composition of the bioglass used in the production of composites may synergistically affect the biological response of the model. In addition, an important factor determining the effectiveness of the biomaterial may be the proportion of chitosan and bioglass in the composite. For CHBG1, CHBG2b, and CHBG3b where the chitosan and bioglass ratio was 1:1, increased cytotoxicity was observed by the direct method. This indicates that there is a need to optimize the amount of chitosan and bioglass in the future. One solution can be a slight reduction in the amount of oxide enriched bioglass to maintain the level of released ions at the level that will still promote cell proliferation.

The toxicological assessment of medical devices should also consider what assays are being used to quantify the cytotoxicity of a material. Examples of quantitative toxicity tests found in the ISO 10993-5:2009 standard are MTT, XTT, and neutral red uptake. Most of the tests mentioned in ISO standard require removal of the culture medium and adding reagents that may precipitate any remaining proteins in the samples. Conducting such tests on the cells seeded directly on the liquid absorbing composites carry the risk of reduced accuracy of the results. Therefore, we suggest using simpler assays, such as WST-1 and LDH, which do not require complicated protocols, and allow to reduce or even eliminate undesirable factors influencing the variability of results. Additionally, tests that quantify the effects observed in different cell compartments support the understanding of the mechanisms of toxicity. Furthermore, determining the toxic effects of the biomaterial on the in vitro model using at least two assays based on different mechanisms of toxicity may be of key importance for the correct cytotoxicity assessment. Another important factor to consider in quantification of cytotoxicity is the physiological state of the cell model, and therefore, a toxicity test including a positive control (e.g., LDH) should be considered essential. Toxicological data presented in many research studies on composites composed of chitosan and bioglass often do not include descriptions of more than one toxicity parameter [28,31,39] or it is not quantitative [26]. Often the growth of control cells which did not have a contact with the material are also not presented or taken into account, which makes it impossible to reliably assess the toxicity of the tested samples [28,39,40].

Another important factor when comparing the toxicity of materials is the method of statistical analysis used. Even extensive studies comparing the effectiveness of different composites do not justify the choice of the applied statistical tests [29,41] or do not provide information about the types of tests used [25]. ANOVA analysis is very popular, but that type of calculation is used for the same quantity of samples and with a normal distribution of data. In most research papers, information about the normal distribution of data is missing [27,29,30,41]. Failure to confirm the normality of the data distribution means that analysis based on parametric tests (ANOVA [42]) and post hoc parametric tests, e.g., Fisher’s least significant difference (LSD) allows for test type 1 error. When assessing the toxicity of composites, this means false-positive results, such as statistically significant differences between the pro-regenerative potential of the tested materials or the biological activity of the material in the following days of experiment.

Based on our study we recommend that selecting only one method of testing should apply only after determining all the parameters related to the production and the most important physical and chemical properties of the product. For all new biocomposites, we strongly suggest using both direct and indirect methods and comparing the results, rather than relying only on one method.

The methodological diversity in the experimental designs describing the toxicity testing of bone regeneration materials suggests the need for the development of uniform guidelines for the determination of cytotoxicity. Despite the ISO 10993-5:2009 standard organizing many aspects of research work, including ones with composites it is important to clarify the details, such as seeding cells on the material or checking the base viability of the cell model.

## Figures and Tables

**Table 1:** Cell lines and methods used for toxicity studies of chitosan-bioglass composites.

Cell Line	Material	Method
**MG-63**—*Homo sapiens* osteosarcoma bone cell line	chitosan and copper (II)—chitosan, chitosan—nano-bioglass	direct [25,26]
**hBMSCs**—human bone marrow stromal cells	chitosan—bioglass, gelatine—chitosan—bioactive nanoceramic	direct [27,28]
**MC3T3-E1**—mouse osteoblastic cell line	chitosan-bioglass and ursolic acid loaded chitosan—bioglass	indirect [29]
**HS-5**—human bone marrow/stroma cells	chitosan, chitosan—bioglass, boronic acid—chitosan—bioglass	direct [30]
**hMSC**—human mesenchymal stem cells	chitosan—bioactive glass and gelatine—chitosan—bioactive glass	direct [31]
**hFOB 1.19**—human primary foetal osteoblasts	chitosan—bioactive glass and gelatine—chitosan—bioactive glass	direct [31]

**Table 2:** Short characteristic of biocomposites used in toxicity testing.

Type of the Bioglass	Bioglass/Polymer Weight Ratio	Composites Designation
P5	1:1	CHBG1
P5Sr2	1:0.5	CHBG2a
	1:1	CHBG2b
	1:2	CHBG2c
P5Zn2	1:0.5	CHBG3a
	1:1	CHBG3b
	1:2	CHBG3c

## Data Availability

The raw/processed data required to reproduce these findings cannot be shared at this time due to legal reasons, the data are also a part of an ongoing study.

## Supplementary Materials

The following supporting information can be downloaded at: https://www.mdpi.com/article/10.3390/polym14214522/s1, Table S1: Results of statistical comparison between pores sizes; Table S2: Results of statistical comparison between proliferation in indirect method assessed with use of WST-1 assay; Table S3: Results of statistical comparison between proliferation in direct method assessed with use of WST-1 assay; Table S4: Results of statistical comparison between effects measured in LDH assay for direct and indirect method; Table S5: Results of statistical comparison between cytotoxicity in indirect method assessed with use of LDH assay; Table S6: Results of statistical comparison between cytotoxicity in direct method assessed with use of LDH assay.
