# Supplementary material for: The Preliminary Assessment of New Biomaterials Necessitates a Comparison of Direct and Indirect Cytotoxicity Methodological Approaches
Source: Polymers (Basel). 2022 Oct 25;14(21):4522. doi: 10.3390/polym14214522 (PMC9657594; doi:10.3390/polym14214522)
Supplement: Supplementary file 1 [file polymers-14-04522-s001.zip › polymers-1940529-supplementary.pdf]

polymers

MDPI

Supplementary Materials: The Preliminary Assessment of New Biomaterials Necessitates a Comparison of Direct and Indirect Cytotoxicity Methodological Approaches

Milena Chraniuk, Mirosława Panasiuk, Lilit Hovhannisyan, Sabina Żołędowska, Dawid Nidzworski, Lidia Ciołek, Anna Woźniak, Zbigniew Jaegermann, Monika Biernat and Beata Gromadzka

Table S1. Results of statistical comparison between pores sizes.

Comparison	Difference statistically significant	*p* value
CHBG2c vs. CHBG3c	No	0.0900
CHBG2c vs. CHBG2a	No	>0.9999
CHBG2c vs. CHBG3a	Yes	<0.0001
CHBG2c vs. CHBG1	No	>0.9999
CHBG2c vs. CHBG3b	Yes	<0.0001
CHBG2c vs. CHBG2b	No	>0.9999
CHBG3c vs. CHBG2a	No	0.4949
CHBG3c vs. CHBG3a	No	>0.9999
CHBG3c vs. CHBG1	No	0.8198
CHBG3c vs. CHBG3b	No	>0.9999
CHBG3c vs. CHBG2b	No	>0.9999
CHBG2a vs. CHBG3a	Yes	<0.0001
CHBG2a vs. CHBG1	No	>0.9999
CHBG2a vs. CHBG3b	Yes	<0.0001
CHBG2a vs. CHBG2b	No	>0.9999
CHBG3a vs. CHBG1	Yes	<0.0001
CHBG3a vs. CHBG3b	No	>0.9999
CHBG3a vs. CHBG2b	Yes	<0.0001
CHBG1 vs. CHBG3b	Yes	<0.0001
CHBG1 vs. CHBG2b	No	>0.9999
CHBG3b vs. CHBG2b	Yes	<0.0001

Table S2. Results of statistical comparison between proliferation in indirect method assessed with use of WST-1 assay.

Comparison	Difference statistically significant	*p* value
CHBG2c vs. CHBG3c	Yes	<0.0001
CHBG2c vs. CHBG2a	Yes	<0.0001
CHBG2c vs. CHBG3a	Yes	0.0002
CHBG2c vs. CHBG1	No	0.1804
CHBG2c vs. CHBG3b	Yes	<0.0001
CHBG2c vs. CHBG2b	No	0.1038
CHBG3c vs. CHBG2a	Yes	<0.0001
CHBG3c vs. CHBG3a	Yes	<0.0001
CHBG3c vs. CHBG1	Yes	<0.0001
CHBG3c vs. CHBG3b	Yes	<0.0001
CHBG3c vs. CHBG2b	Yes	<0.0001
CHBG2a vs. CHBG3a	No	0.0647
CHBG2a vs. CHBG1	Yes	<0.0001
CHBG2a vs. CHBG3b	Yes	<0.0001
CHBG2a vs. CHBG2b	Yes	<0.0001

CHBG3a vs. CHBG1	Yes	0.0001
CHBG3a vs. CHBG3b	Yes	<0.0001
CHBG3a vs. CHBG2b	Yes	0.0001
CHBG1 vs. CHBG3b	Yes	<0.0001
CHBG1 vs. CHBG2b	No	0.1101
CHBG3b vs. CHBG2b	Yes	<0.0001

Table S3. Results of statistical comparison between proliferation in direct method assessed with use of WST-1 assay.

Comparison	Difference statistically significant	*p* value
CHBG2c vs. CHBG3c	No	0.3271
CHBG2c vs. CHBG2a	No	0.1194
CHBG2c vs. CHBG3a	No	0.2247
CHBG2c vs. CHBG1	Yes	<0.0001
CHBG2c vs. CHBG3b	Yes	<0.0001
CHBG2c vs. CHBG2b	Yes	<0.0001
CHBG3c vs. CHBG2a	No	0.2170
CHBG3c vs. CHBG3a	No	0.3398
CHBG3c vs. CHBG1	Yes	<0.0001
CHBG3c vs. CHBG3b	Yes	<0.0001
CHBG3c vs. CHBG2b	Yes	<0.0001
CHBG2a vs. CHBG3a	No	0.2891
CHBG2a vs. CHBG1	Yes	<0.0001
CHBG2a vs. CHBG3b	Yes	<0.0001
CHBG2a vs. CHBG2b	Yes	<0.0001
CHBG3a vs. CHBG1	Yes	<0.0001
CHBG3a vs. CHBG3b	Yes	<0.0001
CHBG3a vs. CHBG2b	Yes	<0.0001
CHBG1 vs. CHBG3b	No	0.2170
CHBG1 vs. CHBG2b	No	0.4207
CHBG3b vs. CHBG2b	No	0.2170

Table S4. Results of statistical comparison between effects measured in LDH assay for direct and indirect method.

Composite	Difference statistically significant	*p* value
CHBG1	Yes	0.0024
CHBG2a	No	0.2773
CHBG2b	Yes	0.0002
CHBG2c	Yes	0.0011
CHBG3a	Yes	0.0002
CHBG3b	Yes	0.0002
CHBG3c	No	0.2773

Table S5. Results of statistical comparison between cytotoxicity in indirect method assessed with use of LDH assay.

Comparison	Difference statistically significant	*p* value
CHBG2c vs. CHBG3c	Yes	<0.0001
CHBG2c vs. CHBG2a	No	0.2884
CHBG2c vs. CHBG3a	No	0.2871
CHBG2c vs. CHBG1	No	0.2105
CHBG2c vs. CHBG2b	No	0.1119
CHBG2c vs. CHBG3b	No	0.0867
CHBG3c vs. CHBG2a	Yes	<0.0001
CHBG3c vs. CHBG3a	Yes	<0.0001

CHBG3c vs. CHBG1	Yes	<0.0001
CHBG3c vs. CHBG2b	Yes	<0.0001
CHBG3c vs. CHBG3b	Yes	<0.0001
CHBG2a vs. CHBG3a	No	0.0658
CHBG2a vs. CHBG1	No	0.4519
CHBG2a vs. CHBG2b	No	0.2884
CHBG2a vs. CHBG3b	No	0.2715
CHBG3a vs. CHBG1	No	0.0502
CHBG3a vs. CHBG2b	Yes	0.0212
CHBG3a vs. CHBG3b	Yes	0.0154
CHBG1 vs. CHBG2b	No	0.5023
CHBG1 vs. CHBG3b	No	0.4519
CHBG2b vs. CHBG3b	No	0.5745

Table S6. Results of statistical comparison between cytotoxicity in direct method assessed with use of LDH assay.

**Comparison**	**Difference statistically significant**	***p* value**
CHBG2c vs. CHBG3c	Yes	0.0005
CHBG2c vs. CHBG2a	Yes	0.0003
CHBG2c vs. CHBG3a	Yes	0.0402
CHBG2c vs. CHBG1	Yes	<0.0001
CHBG2c vs. CHBG2b	Yes	<0.0001
CHBG2c vs. CHBG3b	Yes	<0.0001
CHBG3c vs. CHBG2a	Yes	<0.0001
CHBG3c vs. CHBG3a	Yes	0.0094
CHBG3c vs. CHBG1	Yes	0.0044
CHBG3c vs. CHBG2b	Yes	<0.0001
CHBG3c vs. CHBG3b	Yes	<0.0001
CHBG2a vs. CHBG3a	Yes	<0.0001
CHBG2a vs. CHBG1	Yes	<0.0001
CHBG2a vs. CHBG2b	Yes	<0.0001
CHBG2a vs. CHBG3b	Yes	<0.0001
CHBG3a vs. CHBG1	Yes	<0.0001
CHBG3a vs. CHBG2b	Yes	<0.0001
CHBG3a vs. CHBG3b	Yes	<0.0001
CHBG1 vs. CHBG2b	Yes	<0.0001
CHBG1 vs. CHBG3b	Yes	<0.0001
CHBG2b vs. CHBG3b	Yes	0.0448
